# Human granulocytes undergo cell death via autophagy

**DOI:** 10.1038/s41420-018-0131-9

**Published:** 2018-12-05

**Authors:** Teruyuki Kajiume, Masao Kobayashi

**Affiliations:** 1 Mukainada Child Clinic, Hiroshima, Japan; 2 Department of Pediatrics, Graduate School of Biomedical Sciences, Hiroshima University, Hiroshima, Japan

## Abstract

Mature neutrophils must be quickly removed from inflammatory sites to prevent tissue damage. Neutrophil removal is thought to be accomplished primarily through caspase-dependent apoptosis, which involves several genes of mitochondrial origin. However, mature neutrophils show reduced gene transcription and mitochondrial numbers. We predicted that neutrophils utilize other cell death mechanisms and investigated programmed cell death in human peripheral blood mononuclear cells (MNCs) and polymorphonuclear cells (PMNCs or neutrophil fractions). Unlike MNCs, PMNCs did not undergo DNA fragmentation and were not TUNEL positive, but expressed LC3-II, an autophagy marker. We also found that during differentiation, autophagy inhibitor 3-MA, and not caspase inhibitor zVAD-fmk, prevented segmentation of the nucleus, indicating that these cells undergo autophagy during maturation. Therefore, human neutrophils may undergo spontaneous autophagic cell death rather than apoptosis, during which autophagy may be essential for both maturation and death.

## Introduction

Neutrophils are polymorphonuclear cells (PMNCs) that comprise the first line of defense of the body. They are key players of the innate immune system with major roles in defending against several bacterial and fungal infections. Neutrophils are identified by their specific segmented nucleus and granules storing antimicrobial molecules. While fully functional neutrophils are essential for defending against infections, they must be efficiently removed from inflamed sites to prevent excessive host tissue damage^1,2^. Neutrophils are produced in the bone marrow and have a high turnover rate. Recently, the blood lifespan of neutrophils was reported to be 5.4 days in vivo as compared to ex vivo studies, which estimated their half-life to be 8 h in humans^3^.

Neutrophil death is thought to occur primarily through apoptosis, but also through other mechanisms such as autophagy, necrosis, and a unique death mechanism known as NETosis^4,5^. Programmed cell death is classified into at least three different types^6,7^. Type 1 cell death or apoptosis is caused by DNA fragmentation via a pathway involving members of the caspase family of proteins. Type 2 cell death or autophagy differs from apoptosis in that it involves autophagic vacuoles rather than caspases. Autophagy involves intracellular protein-degradation pathways such as the ubiquitin–proteasome pathway. Membrane-bound LC3-II has also been shown to play an essential role in autophagic cell death and is upregulated during autophagy^8^. Type 3 cell death is similar to necrosis.

Mature neutrophils were suggested to have decreased gene transcription and fewer mitochondria^5,9^. Thus, we hypothesized that granulocytes are unlikely to undergo caspase-mediated apoptosis. In this study, we investigated other mechanisms of programmed cell death in human neutrophils.

## Results

**Fig. 1:**
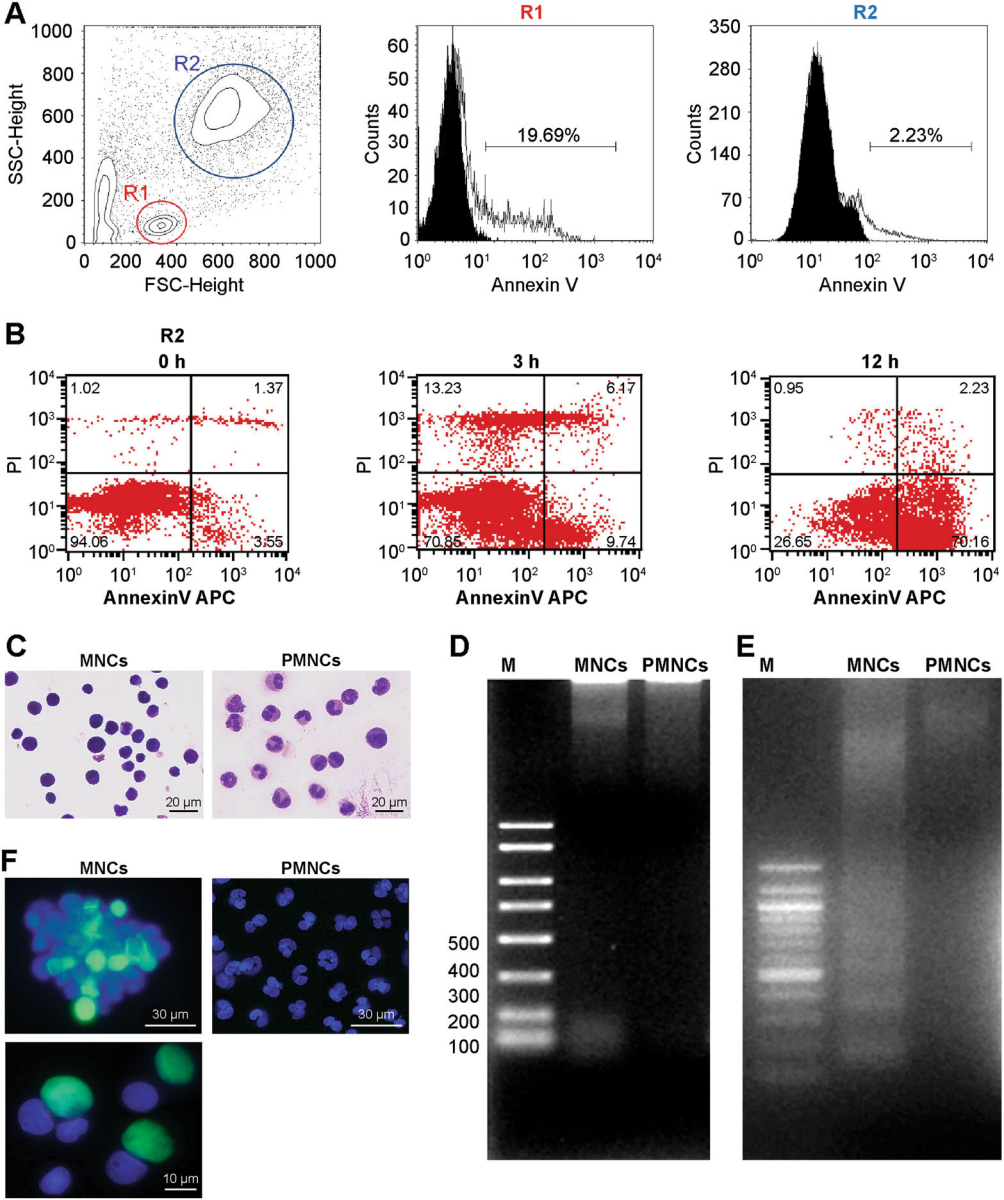
Annexin V and DNA fragmentation assays of human polymorphonuclear cells (PMNCs). **a** Freshly isolated human peripheral white blood cells were analyzed by flow cytometry for annexin V staining. MNCs and PMNCs were gated using forward scatter and side scatter, and annexin V staining was analyzed. **b** Annexin V and PI staining at 0, 3, and 12 h after blood collection in PMNCs. **c** May–Giemsa staining shows the separated MNCs and PMNCs. **d** DNA laddering analyzed immediately after separating MNCs and PMNCs. **e** DNA fragmentation of PMNCs and MNCs analyzed after ultraviolet irradiation for 15 min. “M” indicates the DNA marker. **f** TUNEL assay using separated MNCs and PMNCs; *N* = 6

Annexin V-apoptotic cells and propidium iodide (PI)-positive dead cells were determined in PMNCs by flow cytometry. The analysis was performed immediately after cell isolation at 0 h, or after culturing for 3 or 12 h (Fig. 1a, b). A larger fraction of the annexin V-positive cells were present in the lymphocyte population; however, about 3% of the PMNCs were positive only for annexin V immediately after blood collection. The number of annexin V-positive cells increased after 3 h of culture and majority of the cells (about 70%) were annexin V positive after 12 h in culture.

To detect DNA fragmentation, we first separated mononuclear cells (MNCs) and PMNCs immediately after blood collection by centrifugation. Based on Giemsa staining, MNCs were predominantly lymphocytes, while PMNCs were predominantly neutrophils (Fig. 1c). We were unable to detect DNA fragmentation by DNA laddering analysis (Fig. 1d). Therefore, both MNCs and PMNCs were subjected to ultraviolet irradiation before analyzing DNA laddering. DNA fragmentation was evident in MNCs but not in PMNCs (Fig. 1e).

Next, we analyzed the MNCs and PMNCs by conducting a TUNEL (terminal deoxynucleotidyl transferase (TdT) dUTP nick-end labeling) assay to detect apoptosis without ultraviolet irradiation. Similar to the DNA fragmentation results, while MNCs were found to be TUNEL positive, PMNCs were not (Fig. 1f). At higher magnifications, TUNEL positivity was detected within the nuclei of MNCs.

**Fig. 2:**
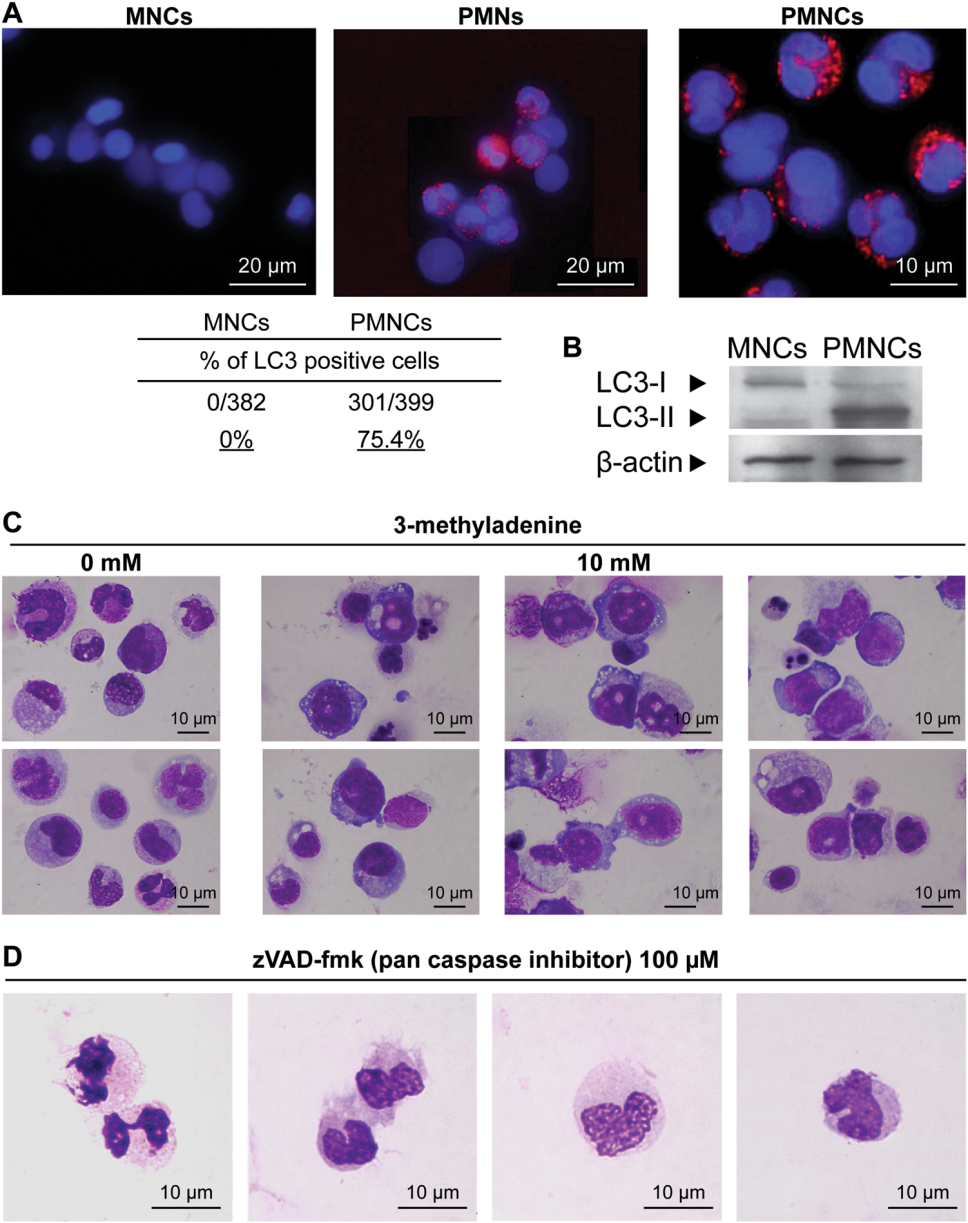
Detection of autophagy in human polymorphonuclear cells. **a** Immunohistochemical staining of MNCs and PMNCs for LC3. The percentage of LC3-positive cells in each fraction was determined by cell counting under a microscope. **b** Western blotting for LC3. **c** Inhibition of autophagy with 3-MA. Human mononuclear cells derived from the bone marrow were incubated with cytokines in the presence or absence of 3-MA at a final concentration of 10 nM. **d** Inhibition of apoptosis with the caspase inhibitor, zVAD-fmk (100 µM); *N* = 6

Next, to examine autophagic cell death, we performed immunohistochemical analysis of LC3 in both MNCs and PMNCs. MNCs were not positive for LC3 but approximately 75% of the PMNCs were positive for LC3 (Fig. 2a, b). Similarly, an LC3-II band (16 kDa) was detected in PMNCs by western blotting (Fig. 2b). LC3-I was cytosolic, whereas LC3-II was membrane bound.

To further determine the role of autophagy in neutrophil survival during neutrophil maturation, we added 3-methyladenine (3-MA), an autophagy inhibitor, to a human myeloid cell differentiation culture assay. 3-MA suppresses the activity of Vps34, a class III phosphoinositide-3 kinase that interacts with beclin 1 during the induction of autophagy^8,10^. Control cells not treated with 3-MA differentiated into mature granulocytes with segmented nuclei. In contrast, the presence of 3-MA (10 mM final concentration) resulted in cells with cytoplasmic granules, but without nucleus segmentation. Maturation arrest was observed at the myeloblast and promyelocyte stages (Fig. 2c). This maturation arrest was not observed in the presence of a caspase inhibitor, Z-Val-Ala-Asp fluoromethylketone (zVAD-fmk; 100 μM final concentration), added in a similar assay (Fig. 2d).

## Discussion

Neutrophils are key players in the innate defense mechanisms. While neutrophil survival and death have been extensively studied, herein, we present a different concept of neutrophil cell death that is mediated primarily via autophagy. Understanding the mechanisms of neutrophil survival and death may aid in understanding their role in pathogenic conditions, particularly in the case of congenital neutropenia.

Similar to previous reports, our results indicate that granulocytes became annexin V positive over the course of time in vitro, suggesting apoptosis^10,11^. However, it is unclear whether annexin V-positive cells are undergoing apoptosis. Annexin V reacts with phosphatidylserine on the inner wall of the cell membrane. Regardless of the type of cell death, the membrane of the cells involved would turn inside-out and collapse, and the cells would become annexin V positive. To directly detect apoptosis, we determined DNA fragmentation and TUNEL positivity in the neutrophils. Previous studies have demonstrated these events in human neutrophils^12–14^, which were typically not observed immediately after isolation, but rather, at later time points in our study. However, we detected DNA fragmentation and TUNEL positivity in MNCs but not in PMNCs. Our results show that human neutrophils may not be constantly undergoing apoptosis and that apoptosis may not be the main primary process responsible for neutrophil turnover.

The LC3 molecule plays an essential role in autophagic cell death. Therefore, autophagy can be analyzed by immunohistochemical analysis to detect LC3^8^. LC3-II is membrane bound and is enriched in autophagic cells. Our results show that approximately 75% of all PMNCs, but not MNCs, stained positively for surface LC3. Collectively, our results suggest that human neutrophils undergo spontaneous autophagic cell death rather than apoptosis. Further, we found that the addition of 3-MA, which suppresses the activity of molecules involved in autophagy^15^, prevents the nuclei of granulocytes from becoming segmented during differentiation, indicating that these cells are undergoing autophagy.

Our findings may also be clinically relevant for cancer treatment. While neutrophils were initially thought to be inert bystanders in cancer conditions, recent studies have indicated that neutrophils may be key players in cancer initiation and progression; they have indeed been shown to be associated with a poor prognosis^16^. Anti-cancer drugs such as 5-fluorouracil (5-FU) have been shown to reduce the neutrophil load in tumor-bearing mice and increase survival times^17^. 5-FU has also been shown to induce autophagy in cancer cell lines^18^, as well as in endothelial cells and cardiomyocytes^19^, and it is possible that 5-FU may be able to induce autophagy in neutrophils under these conditions, thereby eliminating neutrophils, and increasing survival rates in cancer patients. Additionally, other drugs that induce autophagy may also be used to target neutrophils in cancer conditions. For instance, rapamycin and metformin are well-known autophagy-inducing drugs; both have been shown to have anti-tumor effects^20,21^. Further studies are required to understand whether these drugs induce autophagy in neutrophils and whether targeting neutrophils through autophagy can be beneficial for cancer treatment.

Our study had several limitations. We only evaluated apoptosis by DNA fragmentation and TUNEL assays at single time points. These assays should be conducted over longer time periods to detect inherent differences between MNCs and PMNCs. Additionally, caspase expression should be studied to fully understand the apoptotic pathways involved. Further, expression of proteins other than LC3 should be identified for conclusive interpretations for or against autophagy.

Based on our findings, we suggest a different concept in which spontaneous autophagy plays important roles in cell death and differentiation of human neutrophils. However, additional in-depth studies are needed to confirm our results.

## Materials and methods

### Samples

A total of 20 mL of peripheral blood sample (collected four times in EDTA tubes) and 20 mL of bone marrow aspirate were collected from six healthy volunteers recruited from our department after they provided informed consent. This study was approved by the Institutional Review Board at the Hiroshima University School of Medicine (No. 484).

### Detection of apoptosis via annexin V staining

Whole white blood cells were cultured at 37 °C in RPMI-1640 medium (Life Technologies, Carlsbad, CA, USA) without cytokines for 0, 3, or 12 h. The cells were then stained using an Annexin V kit (ANXVKF-100T: Immunostep, Salamanca, Spain) and analyzed by flow cytometry (FACS Calibur, BD Biosciences, San Jose, CA, USA).

### Cell separation

MNCs and PMNCs were separated using Polymorphprep™ (Axis-Shield PoC AS, Oslo, Norway) according to the manufacturer’s protocol. First, 5 mL of blood or bone marrow was layered over 5 mL of Polymorphprep™ and centrifuged at 500 × *g* for 35 min at 22 °C. The MNC and PMNC layers were then collected, and the cells were washed with phosphate-buffered saline. The separated cells were analyzed by Giemsa staining.

### DNA laddering and TUNEL assay

Cells were irradiated for 10 min with a handheld ultraviolet lamp (Analytik Jena, Upland, CA, USA) from a distance of 30 cm. DNA fragmentation was determined using the Apoptotic DNA Ladder Isolation Kit (JM-K170-50; MBL International, Woburn, MA, USA). An In Situ Cell Death Detection Kit (Roche Diagnostics, Mannheim, Germany) was used for the TUNEL assay immediately after cell separation. These kits were used in accordance with the manufacturers’ instructions.

### Immunocytochemical analysis and western blotting to detect LC3 protein

To evaluate the expression of LC3, the cells were fixed with 4% paraformaldehyde. The fixed cells were stained with anti-LC3 antibodies (M152-3; MBL Co. Ltd., Nagoya, Japan). Labeling was performed with the Zenon® labeling kit (Molecular Probes, Inc., Eugene, OR, USA). A fluorescence microscope (BZ-8000; Keyence, Osaka, Japan) was used for observation.

Total protein was extracted and quantified with a protein assay (Bio-Rad, Hercules, CA, USA). Equal amounts of protein were separated by 10% sodium dodecyl sulfate–polyacrylamide gel electrophoresis and transferred onto polyvinyl difluoride membranes (Millipore, Billerica, MA, USA). The membrane was incubated with primary antibodies for LC3 and β-actin (A5316; Sigma-Aldrich, St. Louis, MO, USA) followed with horseradish peroxidase-conjugated anti-mouse IgG (Promega, Madison, WI, USA), and enhanced chemiluminescence (ECL plus; Amersham Life Science, Amersham, UK), as per the manufacturer’s instructions.

### Human myeloid cell differentiation culture assay

Human MNCs derived from bone marrow were incubated in RPMI-1640 medium supplemented with cytokines and 10% fetal calf serum. The following cytokines were used: flt3-ligand (PeproTech, Rocky Hill, NJ, USA) at a final concentration of 20 ng/mL, stem cell factor (Kyowa Hakko Kirin Co., Ltd., Tokyo, Japan) at 100 ng/mL, and granulocyte-colony stimulating factor (Kyowa Hakko Kirin Co., Ltd.) at 100 ng/mL. The cultures were incubated for 7 days under humid conditions with 5% CO_2_ at 37 °C. 3-MA (M9281; Sigma-Aldrich) or zVAD-fmk (C2105; Sigma-Aldrich) was added to some of the cultures. Cells were stained with Giemsa and visualized by light microscopy using a model CX41 light microscope (Olympus, Tokyo, Japan).
